# Outcomes of Traumatic Liver Injuries at a Level-One Tertiary Trauma Center in Saudi Arabia: A 10-Year Experience

**DOI:** 10.3390/life15071138

**Published:** 2025-07-19

**Authors:** Nawaf AlShahwan, Saleh Husam Aldeligan, Salman T. Althunayan, Abdullah Alkodari, Mohammed Bin Manee, Faris Abdulaziz Albassam, Abdullah Aloraini, Ahmed Alburakan, Hassan Mashbari, Abdulaziz AlKanhal, Thamer Nouh

**Affiliations:** 1 Trauma and Acute Care Surgery Unit, Department of Surgery, College of Medicine, King Saud University, P.O. Box 2925, Riyadh 11461, Saudi Arabia; 2 College of Medicine, King Saud University, P.O. Box 2925, Riyadh 11461, Saudi Arabia; 3 Department of Surgery, College of Medicine, King Saud University, P.O. Box 2925, Riyadh 11461, Saudi Arabia; 4 Department of Surgery, Faculty of Medicine, Jazan University, P.O. Box 6809, Jazan 82817, Saudi Arabia

**Keywords:** traumatic liver injury, road traffic accident, motor vehicle crash, ISS, e-FAST, non-operative, operative, mortality, KSA

## Abstract

Traumatic liver injury remains a significant contributor to trauma-related morbidity and mortality worldwide. In Saudi Arabia, motor vehicle accidents (MVAs) are the predominant mechanism of injury, particularly among young adults. This study aimed to evaluate the clinical characteristics, management strategies, and outcomes of patients with liver trauma over a ten-year period at a tertiary academic level-one trauma center. A retrospective cohort study was conducted from January 2015 to December 2024. All adult patients (aged 18–65 years) who sustained blunt or penetrating liver injuries and underwent a pan-CT trauma survey were included. Demographic data, Injury Severity Scores (ISSs), imaging timelines, management approach, and clinical outcomes were analyzed. Statistical analysis was performed using JASP software with a significance threshold set at *p* < 0.05. A total of 111 patients were included, with a mean age of 33 ± 12.4 years; 78.1% were male. MVAs were the leading cause of injury (75.7%). Most patients (80.2%) had low-grade liver injuries and received non-operative management (NOM), with a high NOM success rate of 94.5%. The median time to CT was 55 ± 64 min, and the mean time to operative or IR intervention was 159.9 ± 78.8 min. Complications occurred in 32.4% of patients, with ventilator-associated pneumonia (19.8%) being most common. The overall mortality was 6.3%. Multivariate analysis revealed that shorter time to CT significantly reduced mortality risk (OR = 0.5, *p* < 0.05), while a positive e-FAST result was strongly associated with increased mortality (OR = 3.3, *p* < 0.05). Higher ISSs correlated with longer monitored unit stays (ρ = 0.3, *p* = 0.0014). Traumatic liver injuries in this cohort were predominantly low-grade and effectively managed conservatively, with favorable outcomes. However, delays in imaging and operative intervention were observed, underscoring the requirement for streamlined trauma workflows. These findings highlight the requirement for continuous trauma system improvement, including protocol optimization and timely access to imaging and surgical intervention.

## 1. Introduction

Trauma is a major cause of morbidity and mortality in Saudi Arabia, particularly among children and young adults. In 2016, trauma accounted for 14.4% of in-hospital deaths (6460 out of 44,783), with motor vehicle collisions (MVCs) being the leading cause—responsible for approximately 16 deaths daily in 2019 [1]. Globally, trauma leads to over 5 million deaths annually, and liver injuries represent 15–20% of all abdominal trauma cases, making the liver the most injured solid organ [2]. Blunt trauma, especially from MVCs, is the predominant mechanism, and mortality rates can reach 10% depending on injury severity and system-level factors [3]. These global patterns reflect the local burden in Saudi Arabia and underscore the importance of strengthening liver trauma management systems. Furthermore, liver trauma results from blunt, penetrating, or combined mechanisms. A study conducted by Alferdaus et al. in a tertiary center in Saudi Arabia showed that MVCs caused 70.4% of abdominopelvic trauma, with liver injuries comprising 25.8% of intra-abdominal injuries, followed by spleen injuries (23.1%). In that study, males represented 89.5% of these patients, with a mean age of 30.7 years [4]. Furthermore, another study conducted by Haddad et al. showed that risk factors for increased mortality in trauma patients include head injuries, retroperitoneal hematomas, elevated lactic acid, higher Injury Severity Score (ISS), and older age [5]. These findings highlight the complexity of liver trauma and the requirement for comprehensive assessment and monitoring. According to the World Society of Emergency Surgery (WSES) guidelines, non-operative management (NOM) is the preferred approach for selected liver trauma cases [6]. Surgery is reserved for hemodynamic instability, high-grade lesions, penetrating injuries, and severe associated conditions [7]. Factors predicting NOM failure include high ISS, shock, associated intra-abdominal injuries, peritonitis, and high-grade hepatic vascular injuries seen on CT scans [8,9]. Management decisions should consider injury severity, patient stability, and institutional capabilities, including surgical expertise [10]. Interventional radiology (IR) is increasingly used for volume-responsive patients. A systematic review of 10 studies on Transarterial Embolization (TAE) reported excellent outcomes in hemodynamically unstable patients, advocating for TAE as a distinct “endovascular treatment” category [11]. As IR technology evolves, embolization is expected to remain a key component of liver trauma care [12]. However, discrepancies between guidelines and clinical practice persist. A study of blunt liver trauma found that angioembolization, though strongly recommended, was used in only 0.8% of cases. Surgical management was predominant for severe injuries (64.6%), while NOM was more common for minor injuries (70.9%). The study also reported a mortality rate of 16%, with significant predictors including age, ISS, hemodynamic status, and massive transfusion requirements [13]. Extended focused assessment with sonography in trauma (e-FAST) is the initial modality of choice in blunt abdominal trauma due to its accessibility, though it lacks sensitivity for hollow viscus injuries. Computed tomography (CT) remains the gold standard for diagnosing liver trauma, with clinical and laboratory findings playing a complementary role [14]. Complications from liver trauma range from minor (pleural effusions and bacteremia) to major (bilomas, hematomas, and abscesses), even in patients managed non-operatively [15]. In this cohort, we are presenting a ten-year experience of a level-one trauma center in Riyadh, Saudi Arabia, dealing with traumatic liver injury. Our aim is to assess the clinical profile, interventions, and outcomes of our population.

## 2. Methodology

Here, we present a retrospective cohort study that assesses the clinical profile, interventions, and outcomes of treating traumatic liver injury in patients who presented to our center. To carry out this study, we reviewed all pan-CT trauma survey radiological reports for patients who presented with any grade of traumatic liver injury at the university level-one trauma center, Riyadh, Saudi Arabia, over the period between January 2015 and December 2024. Thus, any patients who presented to our emergency department (ED) with positive e-FAST and severe hemodynamic instability that were taken directly from the ED to the operating room (OR) or the IR suite for surgical or angioembolization intervention without having a pan-CT trauma survey were not included in this study due to the lack of a full detailed trauma registry in our institute. As a result, our study population primarily consisted of hemodynamically stable or stabilized patients who were able to undergo full radiological assessment. This inherently limits our cohort to those with less severe or non-immediately life-threatening injuries, which should be considered when interpreting the overall complication rates and outcomes reported in this study. Consequently, the findings may not fully represent the entire spectrum of liver trauma cases that have presented to our institute in the last 10 years, particularly those with the most severe presentations requiring immediate surgical or radiological intervention. We reviewed patient medical records for demographics (age, gender, and nationality) and the clinical profile of the initial vitals (systolic blood pressure (SBP), heart rate (HR), respiratory rate (RR), oxygen saturation (SpO2%), and Glasgow Coma Scale Score (GCS)). Moreover, e-FAST information was collected, specifying the positive region found during this study. In addition, we calculated the time from patient presentation to the CT scan, and then the time from the CT scan to the final radiological report; these data were uploaded to the system. The management of the patients was classified based on the type of intervention. “Operative management” (OM) was denoted as any patient undergoing exploratory laparotomy or IR angioembolization directly from the ED to the OR or IR suite. “Non-operative management” (NOM) was denoted as any patient undergoing conservative measures to manage liver injuries in the surgical ward, high-dependency unit (HDU), or intensive care unit (ICU). “Failure of non-operative management” was defined as any patient—at any time in the admission process—who required surgical or IR intervention due to liver injuries and was taken to the OR or IR suite after the initial decision of NOM at admission. Nonetheless, management decisions (NOM vs. OM) were made at the discretion of the attending trauma surgeon based on clinical judgment. Factors influencing the decision included hemodynamic stability, imaging findings, grade of liver injury, and the presence of associated intra-abdominal or systemic injuries. No formal institutional protocol was in place during the study period. Furthermore, we documented the time required for patients who received OM to be shifted from the ED to the OR or IR for surgical or angioembolization intervention; this was defined as “Time to OR/IR”. Moreover, the outcomes of the patients included monitored unit (ICU and/or HDU) length of stay (LOS), hospital LOS, complications, overall mortality, 30-day mortality, 30-day ED visit, readmission, and overall complications. In addition, the ISS was calculated for all patients to present the severity of injuries for the population. The inclusion criteria were patients aged 18 to 65 who presented to the ED as trauma patients. Missing data were assessed for patterns and proportions across all variables. For variables with less extensive missing data, a complete case analysis approach was adopted. This study was approved by the Institutional Review Board (IRB) at King Saud University Medical City (approval no: IRB no. E-23-8417). Given the retrospective nature of this study and the use of anonymized patient data, the requirement for informed consent was waived by the IRB. Data were analyzed using JASP software (JASP Team (2024), JASP (Version 0.19.0) [Computer software]), an open-source statistical package that was readily accessible to the research team. JASP was selected due to its ease of use, robust support for both descriptive and inferential tests, and integrated visualization tools, which facilitated the transparent reporting of our findings. Categorical variables were expressed as frequencies by percentages and numbers. The mean and median values with standard deviation (SD) and interquartile range (IQR) were used to measure the central tendency whenever appropriate. The *t*-test and Mann–Whitney test were used for continuous variables with and without normal distribution, respectively. To assess the relationships between continuous variables, correlation analysis was performed using Spearman’s rank-order correlation. Moreover, logistic and linear regression were used to add multivariate analysis to the data. The Shapiro–Wilk test was used to assess the normality of the data. A *p*-value less than 0.05 was considered statistically significant. This study was reported in accordance with the STROBE (Strengthening the Reporting of Observational Studies in Epidemiology) guidelines for observational cohort studies (Table S1).

## 3. Results

**Figure 1:**
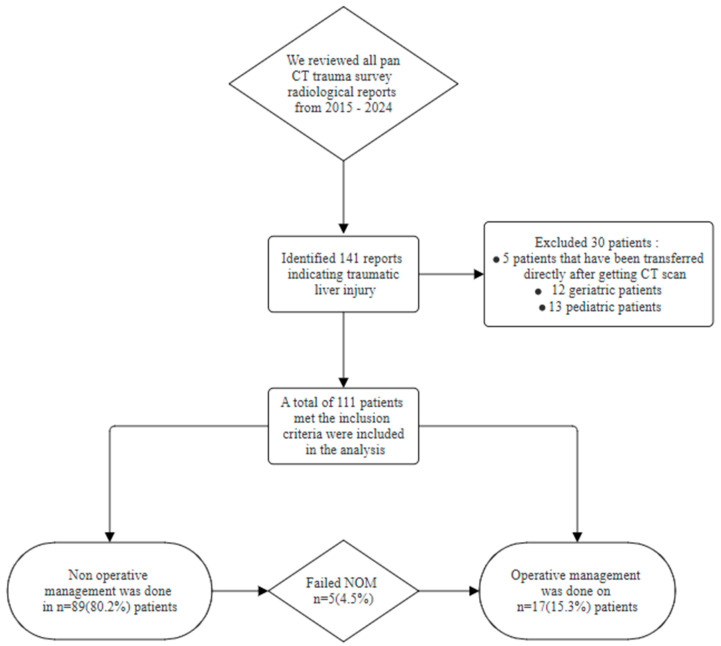
Flowchart of petition selection and management plans.

Of all the pan-CT radiological reports over the ten-year period, we identified 141 patients; however, only 111 patients met the inclusion criteria, as 13 patients were pediatric, 12 patients were geriatric, and 5 patients were transferred after the CT scan was conducted in our center (Figure 1).

In our analysis, the majority of patients were Saudi (75; 67.6%). Furthermore, males dominated the population with 82 (78.1%) against 23 (21.9%) females. Our trauma patients were young adults with a mean age of 33 ± 12.4 years. All of our population had blunt trauma, with only one case of penetrating injury due to a stab wound. Moreover, the majority of admissions were due to MVAs (84; 75.7%), but we had 11 cases that were “Pedestrian vs. Car”, 12 cases due to “Fall from height (more than 6 m)”, and three cases of “Motorcycle crash”. Nonetheless, the mean GCS at arrival was 11.9 ± 4.1, and only 34 (30.6%) patients lost consciousness at the scene. The mean shock index was 1.1 ± 0.4, and our population had a mean ISS of 27 ± 14. Moreover, vital signs were collected at arrival. The mean HR of the population was 105.3 ± 24.4 bpm, RR was 21.8 ± 5.8 breaths per minute, SBP was 114.7 ± 28.7 mmHg, and the mean temperature was 36.6 ± 0.5 °C (Table 1).

**Figure 2:**
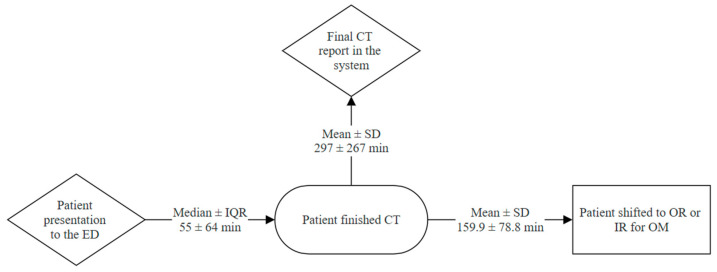
Trauma patient workflow from ED presentation to operative management.

**Figure 3:**
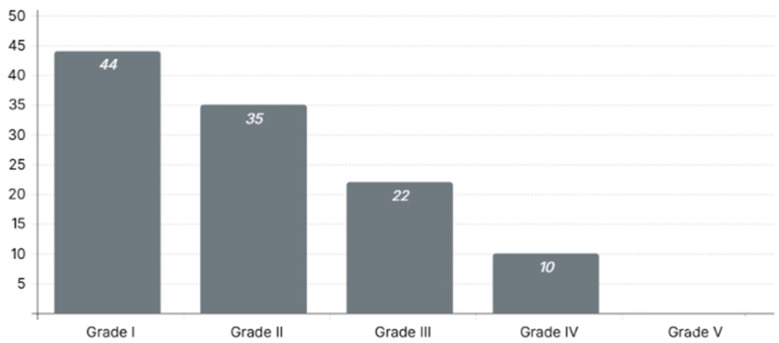
Distribution of liver injury grades among trauma patients.

As shown in Table 2, e-FAST was conducted for the majority of our cohort (95.5%), and only five patients (4.5%) did not have a documented e-FAST in the charts. e-FAST was found to be positive in 38 patients (34.2%) in our cohort, and only 28 patients (25.2%) had a positive e-FAST in Morrison’s pouch, and our data showed that less than half of our population (40%) were intubated in the ED; moreover, most of the admissions were monitored units (ICU and/or HDU) (42.3%). The median time to CT was 55 ± 64 min (~1 h) and the median time to obtain a full detailed report was 297 ± 267 min (~5 h); however, in our center, the critical findings are always conveyed to the trauma team during the CT scan, but this was poorly documented, and thus it was not collected. Moreover, the average time for patients who underwent OM to reach the OR or IR from the ED was 159.9 ± 78.8 min (~3 h) (Figure 2). Nonetheless, most patients sustained low-grade liver injuries, with Grade I injuries being the most common, observed in 44 patients (39.6%), followed by Grade II in 35 patients (31.5%) and Grade III in 22 patients (19.8%). Higher-grade injuries were less frequent, with Grade IV occurring in 10 patients (9%) and no cases of Grade V injuries reported in the cohort (Figure 3). Furthermore, regarding the liver injuries, 89 (80.2%) of the patients had low-grade liver injuries and underwent successful NOM, while 17 patients (15.3%) underwent OM, and only 5 (4.5%) patients failed NOM. Moreover, among the patients who initially received NOM, five cases experienced failed NOM, all of which involved Grade III liver injuries. These patients were initially hemodynamically stable and admitted for conservative treatment; however, they subsequently developed clinical deterioration characterized by hemodynamic instability. As a result, three patients required operative intervention, while the remaining two underwent angioembolization via IR. All five patients responded well to the interventions, were discharged in stable condition, and did not return with any complications during the follow-up period.

Furthermore, the median hospital LOS of our population was 13 ± 15.5 days, with a median duration of 3 ± 10 days in monitored units (ICU and/or HDU). Moreover, 96 patients (86.5%) were discharged home with instructions and follow-up assessment in our clinic; however, only 6 (5.4%) were referred to rehabilitation facilities. Of the total number of patients included in this study, 36 (32.4%) experienced one or more complications. The most frequently observed complication was ventilator-associated pneumonia (VAP), which was reported in 22 patients (19.8%). Catheter-associated urinary tract infection (CAUTI) occurred in eight cases (7.2%), while surgical site infection (SSI) and sepsis were each noted in seven patients (6.3%). Bleeding was reported in six patients (5.4%), pneumothorax in three patients (2.7%), and pleural effusion in two patients (1.8%). Additionally, 12 patients (10.8%) experienced other complications, which included one case of acute kidney injury (AKI), AKI and hepatitis, bile leak and brachial deep vein thrombosis (DVT), cerebrospinal fluid (CSF) leak, DVT, hepatic abscess, hepatitis, hypothermia, peritonitis, and renal vein thrombosis. Moreover, the overall mortality in our population reached 6.3%, but the 30-day mortality was 8.1%. A total of 13 patients visited the ED within 30 days of discharge, and only 10 patients required readmission (Table 3).

In the univariate analysis examining mortality in relation to CT scan timing, no statistically significant differences were observed for either the “Time to CT” or the “Time to CT scan report,” with *p*-values of 0.3 and 0.6, respectively. Similarly, the results of the e-FAST examination showed no significant association with the time taken for patients to undergo a CT scan (*p* = 0.2) or receive the final CT report (*p* = 0.2). Furthermore, no significant association was found between e-FAST results and mortality (*p* = 0.5), indicating that e-FAST alone did not predict patient survival outcomes in the univariate setting. Additionally, we compared ISSs between patients who were readmitted and those who were not, after excluding all mortalities. Interestingly, patients who were not readmitted within 30 days had significantly higher ISSs (27.4 ± 11.2) compared with those with complications (20 ± 7.2), with the difference barely reaching statistical significance (*p* = 0.046). Though this relationship was significant, the readmitted subgroup was small, with only 10 patients readmitted within 30 days compared with 92 patients who were not, which affected the statistical power of the analysis. However, in the multivariate logistic regression analysis incorporating e-FAST, Morison’s pouch findings, ISS, and time to CT scan, a shorter time to CT scan was significantly associated with reduced odds of mortality (OR = 0.5; 95% CI [−1.97, −0.28]; *p* < 0.05). In contrast, a positive e-FAST result was strongly associated with increased mortality risk (OR = 3.3; 95% CI [1.24, 8.78]; *p* < 0.05). While a management plan involving direct intervention was linked to a 26% reduction in mortality (OR = 0.74; 95% CI [0.28, 1.97]; *p* = 0.30), this association did not reach statistical significance. Conversely, failure of conservative management was associated with a 4.5-fold increase in mortality odds (OR = 4.5; 95% CI [0.5, 3.07]; *p* = 0.4), though this finding also lacked statistical significance. In terms of injury severity, a statistically significant positive correlation was observed between ISS and total monitored days. Spearman’s rho confirmed this association with ρ = 0.3 and *p* = 0.0014, indicating that higher ISS values were moderately associated with longer monitored hospital stays.

## 4. Discussion

Although the most common intra-abdominal organ to be injured in abdominal blunt trauma is the spleen [16], traumatic liver injuries are more likely to be fatal and more difficult to manage. In the current study, we investigate 111 low-grade traumatic liver injury patients from a tertiary academic level-one trauma center over the span of 10 years to understand patient outcomes in different management strategies and the factors affecting them. Our population was mainly composed of young adults (30 ± 14 years), as those are usually the most common age group affected by trauma [17]. The majority of our cohort presented after MVAs, with only one patient presenting due to a penetrating injury, which could be attributed to the selection methodology of lower-grade traumatic liver injury that we employed in our cohort, as penetrating traumatic liver injuries are usually more severe but less common [18,19]. The median ISS in our population classifies the severity of the injuries as “Very severe” [20], which was associated with a high shock index of (1.2 ± 0.4). However, this was not reflected in the liver injury grades; the majority of cases were low-grade injuries, with Grade I being the most reported in our cohort (*n* = 44, 39.6%). This result could be due to the patients presenting with additional injuries, including fractures and traumatic brain injuries (TBIs), causing a high ISS in our cohort with the lowest grades of traumatic liver injuries [21]. Moreover, most of our population received e-FAST as an adjuvant for the ATLS protocol [22], which assists in earlier detection of intra-abdominal hemorrhage and prompts faster decision-making in shifting the patient into the OR, especially if associated with hemodynamic instability. Although it can be very effective in cases of major trauma, e-FAST is not always positive in Morrison’s pouch, indicating liver injury, as other locations could be positive, indicating general intra-abdominal injury [23]. Therefore, e-FAST is not effective for low-grade liver injuries or specific deep injuries that ultrasound cannot detect; thus, CT scans are still the gold standard in assessing intra-abdominal solid organ injuries [24]. Although our study was unable to calculate specific false-positive or false-negative rates for e-FAST due to documentation limitations and the lack of standardized reporting templates during the study period, the literature provides robust insight into the diagnostic performance of this modality. e-FAST has been shown to offer high diagnostic accuracy in the context of trauma, with pooled sensitivity estimates ranging from 63% to 99% and specificity from 94% to 100% across multiple studies [25,26]. In particular, e-FAST remains a valuable tool for rapidly detecting intra-abdominal free fluid at the bedside, especially in resource-limited or time-sensitive environments. However, its sensitivity in detecting liver-specific traumatic injuries is notably lower—around 57.5% when all solid organ injuries are considered—while specificity remains high, often exceeding 97% [25]. The diagnostic strength of e-FAST increases with the presence of substantial hemoperitoneum, making it more reliable as a “rule-in” rather than “rule-out” tool in hemodynamically stable patients with minor injuries. These findings highlight the importance of integrating e-FAST with clinical assessment and other imaging modalities. We recommend that future prospective studies implement structured and standardized documentation for e-FAST to allow for more accurate evaluations of its diagnostic utility within local clinical settings. Furthermore, based on multiple guidelines and international recommendations, the pan-CT trauma survey should be conducted in less than 60 min following the patient’s presentation to the trauma bay [27,28]. Our cohort had a median pan-CT trauma survey time of 55 min, with an IQR of 64 min and much longer wait times to receive the full radiological report in the electronic system. Such delays could be attributed to many factors, including the initial implementation of trauma care and the undeveloped protocols and workflow in our institute [29,30]. Furthermore, the installation of a CT scan in the ED with an entire designated team to run the facility could significantly assist in ensuring the application of the best trauma care possible for the presenting patient [31]. In our institute, the CT scan was installed in the ED in 2018; thus, this could be another factor affecting the time to receive the pan-CT trauma survey. Trauma care in Saudi Arabia has historically faced challenges stemming from a fragmented system and uneven distribution of resources. Advanced trauma services have largely been concentrated in a few major tertiary centers, particularly in metropolitan areas such as Riyadh, while peripheral regions often lack access to critical diagnostics such as computed tomography (CT) imaging [4,32]. These limitations have influenced clinical decision-making, often requiring trauma teams to rely on less sensitive bedside tools such as e-FAST and clinical assessment alone. Although valuable, these alternatives may overlook subtle or non-hemorrhagic injuries, potentially delaying definitive intervention in patients with occult trauma [4,32]. At the national level, only a limited number of centers meet level-one trauma criteria and maintain comprehensive trauma registries, while many facilities lack standardized referral pathways and essential infrastructure [4]. These systemic disparities highlight the importance of developing a coordinated, tiered trauma care model. Equitable access to imaging capabilities such as CT should be prioritized across all regions to support timely, evidence-based decision-making. Addressing these gaps is critical not only for improving patient outcomes but also for guiding future planning and resource allocation in the development of a robust national trauma system [4,33]. Moreover, in our population, the mean time to take patients who required OM to the OR or IR suite averaged around 3 h. This delay is due to multiple factors that could affect patient outcomes, including delays in the CT scan access process, poor prehospital triage [34], late trauma team involvement [35], or trauma team delays pertaining to the decision to take a patient into the OR [36]. Based on clinical guidelines and multiple studies, the time to transfer patients to the OR from the trauma bay and receive a pan-CT trauma survey is less than 60 min [37]. Moreover, we had a 94.5% success rate in treating low-grade traumatic liver injuries with NOM, as only 4.5% of our cohort had failed NOM, as this is the prevailing technique in treating low-grade traumatic liver injuries [15]. Moreover, our complication rate during admission was 32.4% of the total population, with the most common complication being VAP, followed by CAUTI. In our cohort, we had 22 patients develop VAP as a complication during their admission. This could be attributed to the lack of application of ventilator protective techniques [38] or to the high rate of intubations in the ED (*n* = 42, 40%) in our cohort, though the current evidence suggests that there is no significant association between ED intubation and development of VAP [39]. In contrast, insertion of a Foley catheter in the ED as an adjuvant to the ATLS protocol could be associated with increased risk of CAUTI, especially if the Foley insertion was not necessary [40].

## 5. Limitations

This study has several limitations that should be acknowledged. First, its retrospective design is inherently limited by the quality and completeness of medical records, with issues such as missing data and poorly documented critical findings during CT scans potentially impacting the results. This study was conducted at a single academic tertiary care level-one trauma center in Riyadh, and as such, the findings may not be generalizable to other healthcare institutions or regions within Saudi Arabia, especially those with different resource levels, trauma care systems, or referral patterns. This is particularly important in a country with diverse healthcare access and infrastructure. The small sample size of 111 patients may also limit the statistical power to detect subtle differences or associations, particularly in rare outcomes such as mortality. There is also potential selection bias, as this study excluded geriatric trauma patients, limiting applicability to older populations. Furthermore, important risk modifiers such as comorbidities, prehospital care variables, and injury severity scoring were not explored in depth, potentially omitting key contributors to outcome variability. Delays in obtaining CT scans were assessed; however, poor documentation and a lack of statistical significance limited meaningful conclusions. While documentation of communication of critical CT findings to the trauma team was inconsistent—preventing a direct evaluation of this important workflow element—our study did include the time from ED arrival to CT acquisition as a surrogate measure to partially capture imaging-related delays. Nevertheless, limitations in timestamp accuracy and missing data precluded robust analysis of time to CT report or radiology communication intervals. Underreporting of failed non-operative management and minimal exploration of e-FAST data further constrain the depth of the clinical analysis. The absence of a control or comparison group and the exclusion of pediatric and geriatric patients reduce the contextual breadth of the findings. Finally, the single-center design inherently limits external validity. Future research should aim for multicenter collaboration, increased sample sizes, inclusion of broader age groups, and prospective designs with standardized documentation protocols to enhance generalizability and inform national trauma care strategies.

## 6. Conclusions

This study provides valuable insight into the clinical profile and outcomes of traumatic liver injuries in Saudi Arabia, highlighting the predominance of motor vehicle accidents among young adult males. Most injuries in our cohort were low-grade and successfully managed non-operatively. Importantly, high ISS and positive e-FAST findings were identified as significant predictors of increased mortality. While delays in imaging and operative intervention were noted, the overall mortality rate remained within expected ranges, underscoring the importance of timely diagnosis and organized trauma care. Future efforts should focus on optimizing trauma workflows and institutional protocols to further improve patient outcomes.

## Figures and Tables

**Table 1:** Population demographics and vitals upon presentation.

Nationality, *n* (%)	Non-Saudi, 36 (32.4%), vs. Saudi, 75 (67.6%)
Gender, *n* (%)	Male, 87 (78.4%), vs. Female, 24 (21.6%)
Age median (±IQR)	30 ± 14 years
Admission diagnosis, *n* (%)	MVAs, 84 (75.7%)
	Pedestrian vs. Car, 11 (9.9%)
	Fall, 12 (10.8%)
	Motorcycle crash, 3 (2.7%)
	Stab wound, 1 (0.9%)
BMI, *n* (±SD)	27.4 ± 6 kg/m^2^
Temperature, *n* (±SD)	36.6 ± 0.5 °C
HR, *n* (±SD)	105.3 ± 24.4 bpm
RR, *n* (±SD)	21.8 ± 5.8 breaths per minute
SBP, *n* (±SD)	114.7 ± 28.7 mmHg
DBP, *n* (±SD)	70 ± 18.7 mmHg
SpO2%, *n* (±SD)	96.7 ± 6.5%
Shock index, *n* (±SD)	1.1 ± 0.4
ISS median (±IQR)	27 ± 14
GCS at arrival, mean (±SD)	11.9 ± 4.1
Loss of consciousness, *n* (%)	Yes, 34 (30.6%)

**Table 2:** e-FAST results, durations related to CT scan, and grades of liver injury.

e-FAST, *n* (%) *	Positive, 38 (34.2%)
Morison pouch, *n* (%) *	Positive, 28 (25.2%)
Peri Splenic recess, *n* (%) *	Positive, 7 (6.3%)
Lung, *n* (%) *	Positive, 3 (2.7%)
Subxiphoid window, *n* (%) *	Negative, 104 (93.7%)
Douglas pouch, *n* (%) *	Positive, 7 (6.3%)
Time to CT median (±IQR)	55 ± 64 min
Time to CT report median (±IQR)	297 ± 267 min
Time to OR/IR, mean (±SD)	159.9 ± 78.8 min
Intubation in ED, *n* (%)	Yes, 42 (40%)
Monitored unit admission before intervention, *n* (%)	Yes, 47 (42.3%)
Monitored unit admission after intervention, *n* (%)	Yes, 44 (39.6%)
Liver injury grade, *n* (%)	Grade I, 44 (39.6%)
	Grade II, 35 (31.5%)
	Grade III, 22 (19.8%)
	Grade IV, 10 (9%)
	Grade V, 0 (0%)
Management, *n* (%)	NOM, 89 (80.2%)
	OM, 17 (15.3%)
	Failure of NOM, 5 (4.5%)

* Indicates missing data.

**Table 3:** Patient outcomes following liver injury.

Hospital LOS median (±IQR)	13 ± 15.5 days
Monitored unit LOS median (±IQR)	3 ± 10 days
Disposition, *n* (%)	Home, 96 (86.5%)
	Rehabilitation, 6 (5.4%)
	Mortem, 9 (8.1%)
Complications, *n* (%)	Total complications, 36 (32.4%)
	VAP, 22 (19.8%)
	SSI, 7 (6.3%)
	CAUTI, 8 (7.2%)
	Sepsis, 7 (6.3%)
	Pneumothorax, 3 (2.7%)
	Pleural effusion, 2 (1.8%)
	Bleeding, 6 (5.4%)
	Other, 12 (10.8%)
Total mortality, *n* (%)	9 (8.1%)
Mortality in 30 days, *n* (%)	7 (6.3%)
ED visit within 30 days, *n* (%)	13 (11.7%)
Readmission within 30 days, *n* (%)	10 (9%)

## Data Availability

The data sets generated and/or analyzed during the current study are not publicly available but are available from the corresponding author upon reasonable request.

## Supplementary Materials

The following supporting information can be downloaded at: https://www.mdpi.com/article/10.3390/life15071138/s1, Table S1: STROBE Statement—checklist of items that should be included in reports of observational studies.
