# Supplementary material for: Outcomes of Traumatic Liver Injuries at a Level-One Tertiary Trauma Center in Saudi Arabia: A 10-Year Experience
Source: Life (Basel). 2025 Jul 19;15(7):1138. doi: 10.3390/life15071138 (PMC12299793; doi:10.3390/life15071138)
Supplement: Supplementary file 1 [file life-15-01138-s001.zip › life-3637578-supplementary.pdf]

STROBE Statement—checklist of items that should be included in reports of observational studies

	Item No.	Recommendation	Page No.	Relevant text from manuscript
**Title and abstract**	1	(*a*) Indicate the study's design with a commonly used term in the title or the abstract	1	Outcomes of Traumatic Liver Injuries at a Level One Tertiary Trauma Center in Saudi Arabia: A 10-Year Experience
		(*b*) Provide in the abstract an informative and balanced summary of what was done and what was found	2-3	Traumatic liver injury remains a significant…
**Introduction**				
Background/rationale	2	Explain the scientific background and rationale for the investigation being reported	4	Trauma is a major cause of morbidity….
Objectives	3	State specific objectives, including any prespecified hypotheses	5	In this cohort we are presenting…
**Methods**				
Study design	4	Present key elements of study design early in the paper	6	Here we present a retrospective cohort
Setting	5	Describe the setting, locations, and relevant dates, including periods of recruitment, exposure, follow-up, and data collection	6	To do that, we have reviewed all pan CT trauma survey
Participants	6	(*a*) *Cohort study*—Give the eligibility criteria, and the sources and methods of selection of participants. Describe methods of follow-up<br>*Case-control study*—Give the eligibility criteria, and the sources and methods of case ascertainment and control selection. Give the rationale for the choice of cases and controls<br>*Cross-sectional study*—Give the eligibility criteria, and the sources and methods of selection of participants	6	Thus any patients who presented to our emergency department (ED) with positive e-FAST and severe hemodynamic
		(*b*) *Cohort study*—For matched studies, give matching criteria and number of exposed and unexposed<br>*Case-control study*—For matched studies, give matching criteria and the number of controls per case	-	-
Variables	7	Clearly define all outcomes, exposures, predictors, potential confounders, and effect modifiers.	6	Then we reviewed patient

		Give diagnostic criteria, if applicable		medical records for demographics (age, gender, and nationality), clinical
Data sources/ measurement	8*	For each variable of interest, give sources of data and details of methods of assessment (measurement). Describe comparability of assessment methods if there is more than one group	6	To do that, we have reviewed all pan CT trauma survey
Bias	9	Describe any efforts to address potential sources of bias	-	-
Study size	10	Explain how the study size was arrived at	6	To do that, we have reviewed all pan CT trauma survey

Continued on next page

Quantitative variables	11	Explain how quantitative variables were handled in the analyses. If applicable, describe which groupings were chosen and why	6	Then we reviewed patient medical records for demographics (age, gender, and nationality), clinical
Statistical methods	12	(*a*) Describe all statistical methods, including those used to control for confounding	7	Data were analyzed using JASP software (JASP Team (2024). JASP (Version 0.19.0)[Computer software]),
		(*b*) Describe any methods used to examine subgroups and interactions	8	To assess the relationships between continuous variables
		(*c*) Explain how missing data were addressed	7	Missing data were assessed for patterns and proportions across all
		(*d*) *Cohort study*—If applicable, explain how loss to follow-up was addressed<br>*Case-control study*—If applicable, explain how matching of cases and controls was addressed<br>*Cross-sectional study*—If applicable, describe analytical methods taking account of sampling strategy	-	-
		(*e*) Describe any sensitivity analyses	8	A p-value less than 0.05
**Results**				
Participants	13*	(a) Report numbers of individuals at each stage of study—eg numbers potentially eligible, examined for eligibility, confirmed eligible, included in the study, completing follow-up, and analysed	9	Of all the pan CT radiological reports over the period of ten years we have had 141 patients
		(b) Give reasons for non-participation at each stage	9	Of all the pan CT radiological reports over the period of ten years we have had 141 patients
		(c) Consider use of a flow diagram	21	***Figure 1 : Flowchart of petitions selection and management plans***
Descriptive data	14*	(a) Give characteristics of study participants (eg demographic, clinical, social) and information on exposures and potential confounders	9	In our analysis the majority of patients were Saudi 75(67.6%).
		(b) Indicate number of participants with missing data for each variable of interest	9	e-FAST was done for the majority of our cohort (95.5%)….
		(c) *Cohort study*—Summarise follow-up time (eg, average and total amount)	-	-
Outcome data	15*	*Cohort study*—Report numbers of outcome events or summary measures over time	*9*	In our analysis the majority of

				patients were Saudi 75(67.6%). Furthermore, males dominated the population with 82 (78.1%) against females 23(21.9%)….
		Case-control study—Report numbers in each exposure category, or summary measures of exposure		
		Cross-sectional study—Report numbers of outcome events or summary measures		
Main results	16	(*a*) Give unadjusted estimates and, if applicable, confounder-adjusted estimates and their precision (eg, 95% confidence interval). Make clear which confounders were adjusted for and why they were included	-	-
		(*b*) Report category boundaries when continuous variables were categorized	-	-
		(*c*) If relevant, consider translating estimates of relative risk into absolute risk for a meaningful time period	-	-

Continued on next page

Other analyses	17	Report other analyses done—eg analyses of subgroups and interactions, and sensitivity analyses	11	In the univariate analysis examining mortality in relation to CT s
**Discussion**				
Key results	18	Summarise key results with reference to study objectives	12	Although the most common intra-abdominal organ to be injured in abdominal blunt trauma is the spleen [15], traumatic liver injuries are more likely to be fatal and more difficult to manage. In the current study we are presenting 111
Limitations	19	Discuss limitations of the study, taking into account sources of potential bias or imprecision. Discuss both direction and magnitude of any potential bias	14	**Limitations:** This study has several limitations that should be acknowledged. First, its retrospective design is inherently limited by the quality and
Interpretation	20	Give a cautious overall interpretation of results considering objectives, limitations, multiplicity of analyses, results from similar studies, and other relevant evidence	12	**Discussion :** Although the most common intra-abdominal organ to be injured in abdominal blunt trauma is the spleen [15],
Generalisability	21	Discuss the generalisability (external validity) of the study results	15	inally, the single-center design inherently limits external validity. Future research should aim for multicenter collaboration,
**Other information**				
Funding	22	Give the source of funding and the role of the funders for the present study and, if applicable, for the original study on which the present article is based	16	**Funding** This study did not receive funding.

*Give information separately for cases and controls in case-control studies and, if applicable, for exposed and unexposed groups in cohort and cross-sectional studies.

Note: An Explanation and Elaboration article discusses each checklist item and gives methodological background and published examples of transparent reporting. The STROBE checklist is best used in conjunction with this article (freely available on the Web sites of PLoS Medicine at http://www.plosmedicine.org/, Annals of Internal Medicine at http://www.annals.org/, and Epidemiology at http://www.epidem.com/). Information on the STROBE Initiative is available at www.strobe-statement.org.
